# SUMOylation of xeroderma pigmentosum group C protein regulates DNA damage recognition during nucleotide excision repair

**DOI:** 10.1038/srep10984

**Published:** 2015-06-04

**Authors:** Masaki Akita, Yon-Soo Tak, Tsutomu Shimura, Syota Matsumoto, Yuki Okuda-Shimizu, Yuichiro Shimizu, Ryotaro Nishi, Hisato Saitoh, Shigenori Iwai, Toshio Mori, Tsuyoshi Ikura, Wataru Sakai, Fumio Hanaoka, Kaoru Sugasawa

**Affiliations:** 1 Biosignal Research Center, Organization of Advanced Science and Technology, Kobe University, Kobe 657-8501, Japan; 2 Graduate School of Science, Kobe University, Kobe 657-8501, Japan; 3 Cellular Physiology Laboratory, RIKEN, Wako 351-0198, Japan; 4 Graduate School of Science and Technology, Kumamoto University, Kumamoto 860-8555, Japan; 5 Graduate School of Engineering Science, Osaka University, Toyonaka 560-8531, Japan; 6 Radioisotope Research Center, Nara Medical University, Kashihara 634-8521, Japan; 7 Radiation Biology Center, Kyoto University, Kyoto 606-8501, Japan; 8 Faculty of Science, Gakushuin University, Tokyo 171-8588, Japan

## Abstract

The xeroderma pigmentosum group C (XPC) protein complex is a key factor that detects DNA damage and initiates nucleotide excision repair (NER) in mammalian cells. Although biochemical and structural studies have elucidated the interaction of XPC with damaged DNA, the mechanism of its regulation *in vivo* remains to be understood in more details. Here, we show that the XPC protein undergoes modification by small ubiquitin-related modifier (SUMO) proteins and the lack of this modification compromises the repair of UV-induced DNA photolesions. In the absence of SUMOylation, XPC is normally recruited to the sites with photolesions, but then immobilized profoundly by the UV-damaged DNA-binding protein (UV-DDB) complex. Since the absence of UV-DDB alleviates the NER defect caused by impaired SUMOylation of XPC, we propose that this modification is critical for functional interactions of XPC with UV-DDB, which facilitate the efficient damage handover between the two damage recognition factors and subsequent initiation of NER.

Genomic DNA in organisms is constantly injured by various agents, which are derived from endogenous as well as exogenous sources. One of the most common environmental genotoxic stressors is ultraviolet (UV) light, which induces characteristic dipyrimidinic DNA photolesions, such as cyclobutane pyrimidine dimers (CPD) and pyrimidine-pyrimidone (6-4) photoproducts (6-4PP). Interfering with replication and transcription, these DNA lesions induce mutations, chromosomal aberrations, and cellular apoptosis, whereas such deleterious effects are counteracted by nucleotide excision repair (NER), a highly versatile DNA repair pathway. In humans, hereditary defects in NER are implicated in several autosomal recessive disorders, including xeroderma pigmentosum (XP); seven XP-related genes, *XPA* through *XPG*, encode proteins that play crucial roles in the human NER pathway. Among these XP gene products, the XPC protein is a key factor involved in DNA damage recognition and initiation of the NER reaction^1^,^2^,^3^.

The XPC protein exists *in vivo* as a heterotrimeric complex, consisting of a human ortholog of yeast Rad23 (RAD23A or B) and centrin-2. Biochemical and structural analyses revealed that the XPC complex is capable of binding specifically to DNA damage sites associated with a relatively large distortion of the DNA duplex by interacting with oscillating normal bases^4^,^5^,^6^. The DNA-bound XPC recruits the general transcription factor IIH (TFIIH) complex, and the two ATPase/helicase subunits, XPB and XPD, locally unwind double-stranded DNA. Thereafter, a fully opened complex, containing 24-30 nucleotide long single-stranded DNA, is formed together with XPA and replication protein A (RPA), which serves as an essential structural platform for the subsequent dual incision by two endonucleases, ERCC1/XPF and XPG. The NER dual incision reactions with defined DNA substrates have been reconstituted *in vitro* with the six purified protein factors (XPC, TFIIH, XPA, RPA, ERCC1/XPF, and XPG)^7^. The resulting single-stranded gap is then filled by DNA polymerases in a PCNA-dependent manner, followed by rejoining of the DNA strands with DNA ligases (for details of the NER molecular mechanism, see recent reviews in Ref. 8 & 9).

Aside from the core part of the NER process, additional protein factors regulate NER *in vivo*. One of such factors is the UV-damaged DNA-binding protein (UV-DDB), a heterodimer consisting of DDB1 and DDB2 (XPE). UV-DDB exhibits strong and specific binding to UV-induced photolesions, CPD and 6-4PP^10^,^11^, and promotes recruitment of XPC^12^,^13^,^14^. Since only a small DNA helix distortion is induced by CPD, direct recognition by XPC is so difficult that intervention by UV-DDB is important for efficient repair of CPD^15^,^16^. By contrast, both the UV-DDB-dependent and independent damage recognition pathways can process 6-4PP. Structural studies of UV-DDB^17^ and the yeast XPC homolog Rad4^4^ revealed that both factors, when bound to damaged DNA, induce a kink of the DNA duplex, but in the opposite directions. This suggests that XPC and UV-DDB may not be able to bind simultaneously to the same site with a DNA lesion, so that a regulated mechanism ensuring a damage handover from UV-DDB to XPC is required. UV-DDB is part of the Cullin-RING ubiquitin ligase complex containing CUL4 and RBX1 (designated as CRL4^DDB2^)^18^, which is activated upon chromatin binding after UV irradiation of cells and thereby ubiquitinates various proteins including DDB2, XPC and histones^19^,^20^,^21^. Such ubiquitination has been implicated in stability of the damage recognition proteins and in regulation of their functional interactions and chromatin structures to allow initiation of NER. When DDB2 was poly-ubiquitinated *in vitro*, the damaged DNA-binding activity of UV-DDB was completely abolished; by contrast, ubiquitinated XPC was still capable of binding to damaged DNA^20^. Based on this observation, we proposed that the CRL4^DDB2^-mediated ubiquitination is important for an efficient handover of damage from UV-DDB to XPC and initiation of the subsequent NER reaction^22^. More recently, however, we have reported that ubiquitination of DDB2 may not be absolutely necessary for such a damage handover *in vivo*^23^, so that the precise molecular mechanisms underlying damage recognition for NER remain to be understood in more details. Notably, neither UV-DDB nor any ubiquitination factor is essential for the core NER reaction reconstituted *in vitro*^7^,^20^.

Small ubiquitin-related modifier (SUMO) proteins are conjugated to lysine side-chains of other proteins through isopeptide bonds, like ubiquitin. A wide variety of proteins, particularly in the nucleus, have been reported to undergo SUMOylation, so that possible roles of these modifications have been suggested in regulation of nuclear pore functions, transcription, replication, repair, and cell cycle (see recent reviews in Ref. 24 & 25). However, precise functions of SUMOylation have been elucidated at molecular levels only for a small number of target proteins. Here we report that the XPC protein is SUMOylated at multiple sites, and abrogation of the modifications results in impaired NER activity. Our results would provide novel insights to *in vivo* regulatory mechanisms of the DNA damage recognition process including both XPC and UV-DDB.

## Results

### XPC interacts with SUMO-1 and SUMO conjugating enzymes

**Figure 1:**
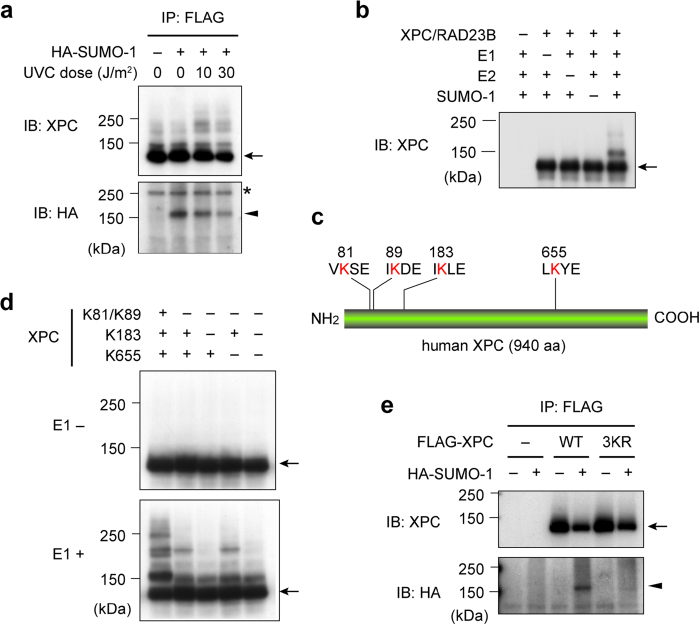
XPC is SUMOylated *in vivo* and *in vitro*. (**a**) Immunoblot analyses of XP4PASV cells stably expressing FLAG-XPC and transfected with a HA-SUMO-1 expression construct (or empty vector as a control). At 8 h post-transfection, the cells were exposed to the indicated doses of UVC and then incubated for 1 h. FLAG-XPC was immunoprecipitated from the cell extracts and subjected to immunoblot analyses (the same blot was sequentially probed with anti-XPC and anti-HA antibodies). The arrow and arrowhead indicate unmodified and SUMOylated XPC, respectively. The asterisk indicates a non-specific antibody reaction. (**b**) Immunoblot analyses of XPC from cell-free SUMOylation reactions performed in the presence of the indicated purified proteins. (**c**) The four putative SUMOylation sites in the human XPC sequence that match the consensus sequence (ψKxE). (**d**) Immunoblot analyses of XPC from cell-free SUMOylation reactions performed with the indicated mutant XPC proteins and with (lower panel) or without (upper panel) E1. The positive (+) and negative (-) symbols indicate the presence of the WT residue (K) and the mutated residue (R), respectively. (**e**) Immunoblot analyses of XP4PASV cells transiently co-expressing HA-SUMO-1 and FLAG-XPC WT or 3KR. Empty vector was used for the lanes labeled “-”. FLAG-XPC was immunoprecipitated and subjected to immunoblot analyses at 48 h post-transfection (the same blot was sequentially probed with anti-XPC and anti-HA antibodies).

To understand functional regulation of XPC *in vivo*, we searched for novel XPC interacting partners by performing a yeast two-hybrid screen of a mouse cDNA library. The positive clones obtained from this screening encoded SUMO-1 and several enzymes involved in SUMO-protein conjugation, including UBE2I (SUMO E2), PIAS1 and PIAS3 (SUMO E3). To test the possibility that XPC undergoes modification by SUMO proteins *in vivo*, hemagglutinin (HA)-tagged SUMO-1 was transiently expressed in an *XPC*-deficient fibroblast cell line (XP4PASV) stably expressing FLAG-tagged XPC at a nearly physiological level. When FLAG-XPC was immunoprecipitated from the cell extracts, SUMOylated XPC was detected with an anti-HA antibody (Fig. 1a). Unlike UV-induced ubiquitination of XPC catalyzed by CRL4^DDB2^, the SUMOylation of XPC occurred regardless of UV treatment (Fig. 1a). The SUMOylation of XPC was also reconstituted *in vitro* using purified protein factors, and immunoblot analyses identified a major and several minor shifted bands of XPC in the reaction (Fig. 1b). In contrast with CRL4^DDB2^-mediated ubiquitination^20^, the level of *in vitro* XPC SUMOylation was only marginally affected by the presence of DNA (Supplementary Fig. S1a). In addition, the DNA-binding activity of XPC was not affected significantly by SUMOylation, regardless of the presence or absence of DNA damage (Supplementary Fig. S1b). Taken together, these data suggest that the SUMOylation of XPC is largely independent of its DNA damage recognition activity.

### XPC is SUMOylated at multiple sites

A consensus amino acid sequence for SUMOylation sites has been proposed (ψKxE; where ψ represents a hydrophobic amino acid)^26^. Because human XPC contains four lysine residues (K81, K89, K183, and K655) that match this consensus sequence perfectly (Fig. 1c), mutant XPC proteins in which these lysines were changed to arginines in various combinations were generated. In cell-free SUMOylation reactions, simultaneous mutations of K81 and K89 abolished the major shifted XPC protein band as well as several minor shifted bands, and the additional mutation of K183 abolished a residual minor band (Fig. 1d). Although SUMOylation of XPC at K655 has been described previously^27^, this site did not seem to be targeted in our cell-free system at a detectable level. Because some degree of XPC protein band shift was still visible even with triple or quadruple lysine mutations, we searched for additional SUMOylation sites in the XPC sequence using the SUMOplot analysis program (Abgent Inc.). Several candidate lysines (such as K113 and K917) were identified, but mutation of these residues failed to abrogate the residual XPC band shift (data not shown). To determine whether the K81, K89, and K183 residues are indeed targets for SUMOylation *in vivo*, wild-type XPC (XPC WT) and a mutant XPC protein containing triple substitutions for these lysines with arginines (XPC 3KR) were fused to an N-terminal FLAG-tag and transiently co-expressed with HA-SUMO-1 in the *XPC*-deficient cell line. Immunoprecipitation of XPC WT and 3KR followed by immunoblotting using an anti-HA antibody revealed that the conjugation of SUMO-1 to FLAG-XPC was almost completely abolished by the triple mutations (Fig. 1e).

### XPC lacking the SUMOylation sites shows NER defects *in vivo*, but not *in vitro*

**Figure 2:**
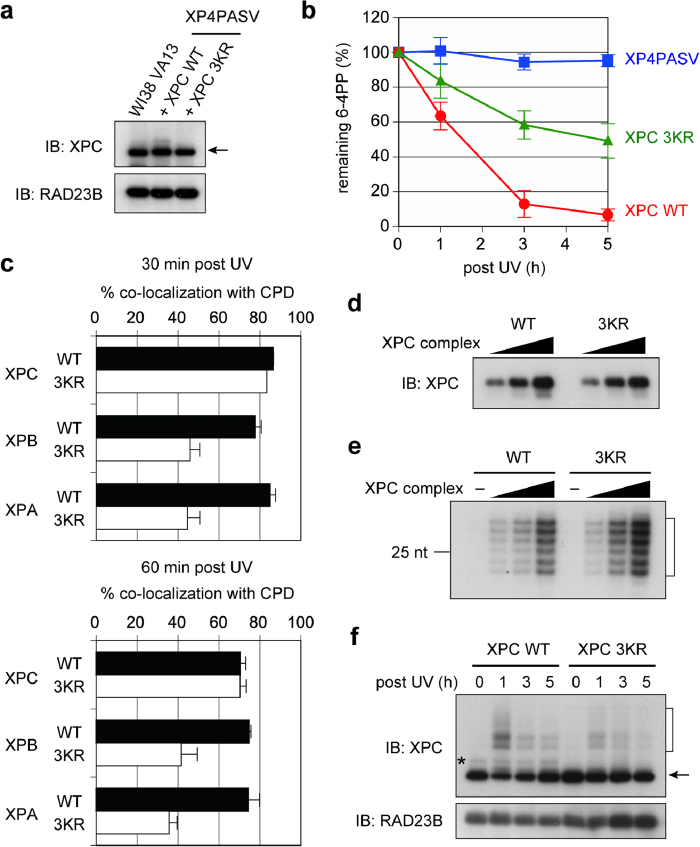
Mutation of major SUMOylation sites in XPC compromises global genome NER and UV-induced ubiquitination of XPC. (**a**) Immunoblot analyses of endogenous XPC levels in normal human fibroblasts (WI38 VA13) and exogenous FLAG-XPC (WT or the 3KR mutant) in stably transfected XP4PASV cells. The same blot was probed with anti-RAD23B antibody as a loading control. (**b**) DNA repair assays of the indicated cell lines following exposure to UVC (10 J/m^2^). The percentages of 6-4PP remaining in the genomic DNA were quantified and plotted as a function of time. The mean values and standard errors were calculated from five independent experiments. (**c**) The transformed cell lines stably expressing XPC WT (solid bar) or 3KR (open bar) were irradiated with UVC (100 J/m^2^) through isopore membrane filters. At 30 or 60 min after irradiation, immunofluorescence staining was performed as shown in Supplementary Fig. S2, and the percentages of CPD foci with detectable accumulation of the indicated NER proteins were measured. The mean values and standard errors were calculated from two independent experiments, in each of which more than 200 CPD foci were analyzed. (**d**) Immunoblots showing that comparable amounts of recombinant XPC WT and 3KR proteins were used in the experiment shown in (**e**). (**e**) *In vitro* NER dual incision assays of recombinant XPC WT and 3KR. The excised oligonucleotides containing a 6-4PP are indicated. (**f**) Immunoblot analyses of UVC-treated (10 J/m^2^) cell lines stably expressing FLAG-XPC (WT or 3KR). After incubation for the indicated times, extracts were prepared and subjected to immunoblot analyses (the same blot was probed sequentially with anti-XPC and anti-RAD23B antibodies). Although HA-DDB2 was ectopically expressed in this experiment to enhance the UV-induced XPC ubiquitination, comparable results were also obtained without DDB2 expression. The asterisk indicates putative SUMOylated XPC bands.

To examine the impact of the 3KR mutations of XPC on NER, transformed XP4PASV cell lines that stably expressed FLAG-XPC WT or 3KR were isolated. For accurate comparison, the expression level of the mutant XPC protein was adjusted to match that of exogenous FLAG-XPC WT in the control cell line and endogenous XPC in normal human fibroblasts (Fig. 2a). After UVC irradiation, the cell line expressing XPC 3KR showed a significantly slower rate of repair of UV-induced 6-4PP than the cells expressing XPC WT (Fig. 2b). To examine which stage in the NER process was affected by the mutations, the cells expressing XPC WT or 3KR were irradiated with UV through isopore membrane filters, and localization of NER factors was visualized with immunofluorescence staining. At 30 min post irradiation, both XPC WT and 3KR proteins similarly co-localized with the major DNA photolesion, CPD (Supplementary Fig. S2). By contrast, recruitment of the downstream NER factors, XPB and XPA, to the UV-damaged areas was significantly compromised in the cells expressing XPC 3KR (Supplementary Fig. S2; see also quantitative data in Fig. 2c).

To test whether the mutations impaired the basal XPC functions as the initiator of NER, the recombinant XPC 3KR protein was tested in cell-free NER assays. When dual incision of 6-4PP by NER was reconstituted with five purified NER factors (TFIIH, XPA, RPA, ERCC1/XPF, and XPG), the specific activity of XPC 3KR was comparable to that of the WT protein, indicating that damage recognition by XPC 3KR and the subsequent repair process were accomplished normally, as long as repair was directly initiated by XPC (Fig. 2d,e). Electrophoretic mobility shift assays confirmed that the 3KR mutations did not alter the DNA binding properties of XPC significantly (Supplementary Fig. S3a and b). Moreover, when SUMO-1 was fused to the N-terminus of XPC 3KR, the observed retardation of 6-4PP repair caused by the 3KR mutations was partially alleviated (Supplementary Fig. S4a and b), whereas *in vitro* SUMOylated XPC showed comparable activities to unmodified XPC in both cell-free NER and damaged DNA-binding assays (Supplementary Fig. S5a-c). Taken together, these results suggest that the SUMOylation of XPC affects a certain auxiliary function of NER, but not the core process, and thereby enhances the repair efficiency of UV-induced photolesions.

### Non-SUMOylated XPC affects functional interactions with UV-DDB

Although UV-DDB is supposed to play a crucial role in efficient recognition of UV-induced photolesions *in vivo*, it is not an essential component of the NER reaction reconstituted *in vitro*. When *in vivo* responses of the XPC protein to UV irradiation were examined, we found that the CRL4^DDB2^-mediated ubiquitination of XPC 3KR was much less pronounced than that of XPC WT (Fig. 2f). Since both ubiquitination and SUMOylation target lysine residues, it can be assumed that the KR substitutions may have abrogated major ubiquitination sites. However, cell-free ubiquitination reactions revealed that both XPC WT and 3KR proteins were ubiquitinated to a similar extent (Supplementary Fig. S3c).

Based on the findings described above, we hypothesized that SUMOylation of XPC may affect its physical and/or functional interaction with UV-DDB. To test this proposal, live cell imaging experiments were performed using transformed XP4PASV cell lines stably expressing enhanced green fluorescent protein (EGFP)-tagged XPC WT or 3KR at nearly physiological levels (Supplementary Fig. S6a). After a pulse of UVC light (emitted from a Hg-Xe lamp and focused through a reflecting objective lens) was applied to a certain area within the nucleus of a cell, accumulation of EGFP-XPC to the irradiated area was monitored via time-lapse observations. EGFP-XPC WT and 3KR were recruited to the UV-damaged sites at comparable rates (Supplementary Fig. S6b). Moreover, pull-down assays with biotinylated DDB2 revealed that neither the 3KR mutations nor SUMO conjugation affected the physical interaction of XPC with UV-DDB (Supplementary Fig. S6c).

**Figure 3:**
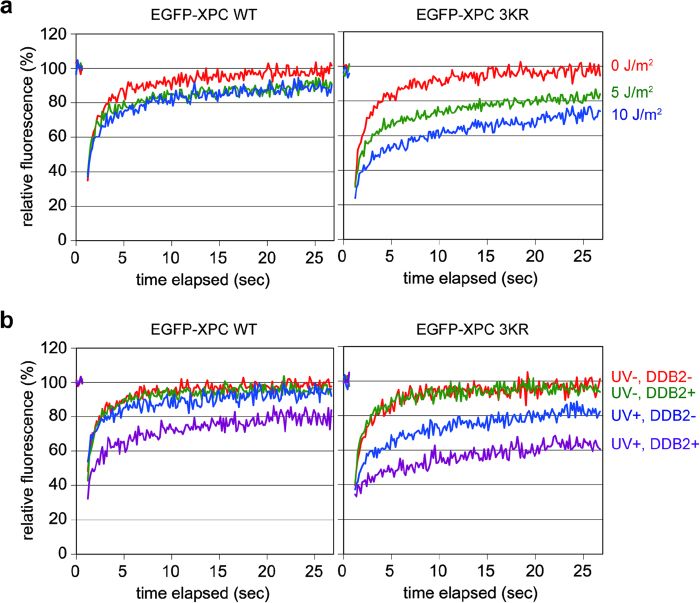
UV-induced and DDB2-dependent immobilization of XPC *in vivo* is augmented by the 3KR mutations. (**a**) FRAP analyses of EGFP-XPC WT (left panel) and EGFP-XPC 3KR (right panel) stably expressed in XP4PASV cells. The cells were globally exposed to UVC at a dose of 0 J/m^2^ (red), 5 J/m^2^ (green), or 10 J/m^2^ (blue), and subjected to FRAP within 30 min. After photobleaching, fluorescence recovery was monitored and plotted as a function of time. At least 20 cells were analyzed for each condition. (**b**) FRAP analyses of EGFP-XPC WT (left panel) and EGFP-XPC 3KR (right panel) in XP4PASV cells transfected with an mCherry-mDDB2 expression construct and treated with 0 J/m^2^ or 10 J/m^2^ UVC at 24 h post-transfection. After FRAP analyses, the data separated into those for cells with and without mCherry fluorescence. At least ten cells were analyzed for each condition.

Next, the *in vivo* mobility of EGFP-XPC was assessed using the fluorescence recovery after photobleaching (FRAP) technique. We have previously reported that the mobility of EGFP-XPC was reduced in a two-step manner upon UV irradiation^28^; the first immobilization caused by relatively low doses of UVC (5-10 J/m^2^) represented UV-DDB-dependent entrapment of XPC at sites with photolesions (mainly 6-4PPs). This UV-induced immobilization of EGFP-XPC was augmented substantially by the 3KR mutations (Fig. 3a). Moreover, when mCherry-tagged DDB2 was transiently overexpressed, this difference was even more pronounced (Fig. 3b), corroborating the notion that the NER defect caused by the XPC 3KR mutations is related to UV-DDB.

### Removal of UV-DDB alleviates the NER defect of XPC 3KR

**Figure 4:**
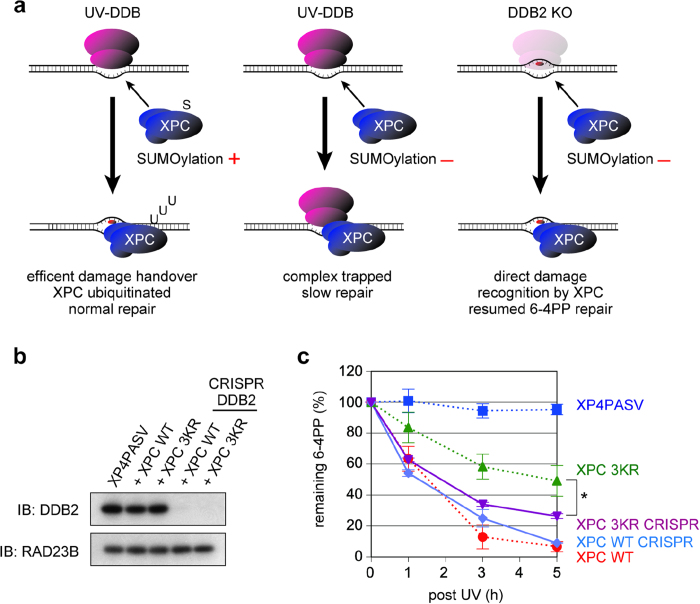
Depletion of DDB2 alleviates the NER defect caused by the 3KR mutations of XPC. (**a**) A model showing the role of XPC SUMOylation in the efficient damage handover from UV-DDB. In the absence of XPC SUMOylation, UV-DDB is retained at the damaged DNA site and NER is interfered. (**b**) Immunoblot analyses of DDB2 expression in the *DDB2*-knockout cell lines stably expressing FLAG-XPC (WT or the 3KR mutant). RAD23B was used as a loading control. (**c**) DNA repair assays of the *DDB2*-knockout cell lines (solid lines) after exposure to UVC (10 J/m^2^). The percentages of 6-4PPs remaining in the genomic DNA were quantified and plotted as a function of time. The mean values and standard errors were calculated from three independent experiments. For comparison with the cell lines containing the intact *DDB2* gene, the data in Fig. 2b are superimposed (dashed lines). Depletion of DDB2 significantly alleviated the slowed 6-4PP repair in the XPC 3KR expressing cells (*P < 0.01).

Collectively, the data described above suggest that XPC 3KR can be recruited normally by UV-DDB bound to photolesions, although the subsequent steps may be interfered, resulting in impaired ubiquitination and prolonged sequestration of XPC, as well as retarded initiation of NER. In the absence of SUMOylation, the efficient damage handover from UV-DDB to XPC may be hampered and UV-DDB may be retained at the damaged sites alongside XPC (Fig. 4a). Based on this model, we hypothesized that the absence of DDB2 would allow XPC 3KR to gain direct access to 6-4PP sites, thereby restoring the damage repair kinetics. To test this possibility, the CRISPR/Cas9 system was used to delete the endogenous *DDB2* gene in the cell lines stably expressing FLAG-XPC WT or 3KR (Fig. 4b). With these *DDB2*-knockout cell lines, we confirmed that the UV-induced ubiquitination of XPC was abrogated (data not shown). In the cells expressing XPC WT, the repair rate of UV-induced 6-4PPs was largely unaffected by depletion of DDB2 (Fig. 4c). This is not surprising because unlike CPD, UV-DDB is known to facilitate repair of 6-4PPs only marginally, especially when relatively high UV doses are used^13^,^29^. By contrast, in the XPC 3KR-expressing cells, depletion of DDB2 significantly alleviated the delay of 6-4PP repair up to a comparable level with the XPC WT cells (Fig. 4c). The residual small difference may be due to a slightly lower expression level of XPC 3KR (~90%) compared with XPC WT in the DDB2 knockout cell lines (data not shown). Therefore, it can be concluded that, in the absence of XPC SUMOylation, UV-DDB bound to UV-damaged DNA sites can block the NER process.

## Discussion

A large number of eukaryotic nuclear proteins have been reported to interact with SUMO proteins and/or undergo SUMOylation. There have been several reports describing XPC SUMOylation^27^,^30^,^31^, one of which suggested K655 as a modification site in human XPC. The presence of two structurally ordered domains was predicted for human XPC; the evolutionarily conserved C-terminal domain is crucial for interactions with DNA and other NER proteins, whereas functions of the N-terminal domain are largely unknown, except for a reported interaction with XPA^32^. Notably, the three major SUMOylation sites (K81, K89, K183) identified in this study are all mapped in the N-terminal domain. Our *in vitro* assays gave rise to multiple species of SUMOylated XPC, among which the most prominent band seemed to depend on the presence of K81 and/or K89. In the pull-down experiments from cell extracts, only a single band of SUMOylated XPC was detectable and, in line with our *in vitro* data, simultaneous mutations of K81 and K89 were sufficient to abolish this band (data not shown). However, transformed XP4PASV cells stably expressing the XPC double mutant did not show a significant defect in NER (data not shown), suggesting that a relatively minor SUMOylation site, K183, may become critical when the two major sites are unavailable. Nevertheless, K655 within the C-terminal domain has only marginal contribution to XPC SUMOylation, if any, in our *in vivo* and *in vitro* analyses. Amino acid sequence alignment and SUMOplot analyses revealed that at least one SUMOylation motif with high probability (score 0.9 or higher) exists in the N-terminal domain of XPC from mouse, chicken, zebrafish, and *C. elegans* (Supplementary Fig. S7). By contrast, the motif corresponding to human K655 is conserved in zebrafish and *C. elegans*, but not in mouse or chicken. Very recently, a mass spectrometric analysis of global SUMOylation in human cells has been reported, indicating SUMOylation of K81 in XPC^33^, further supporting importance of the N-terminal domain as a target of SUMOylation.

Aside from the modification sites, reported nature of XPC SUMOylation has been somewhat contradictory and needed to be clarified. In humans, several SUMO isoforms are expressed; SUMO-2 and SUMO-3 show substantial homology to each other, and their functions are supposed to be distinct from those of SUMO-1^34^. One paper reported that XPC was conjugated to SUMO-1^31^, whereas the other described poly-SUMO-2 chains formed on XPC^30^. Although SUMO-2/3 proteins were not obtained from our two-hybrid screening, SUMO-1 and SUMO-2 behaved indistinguishably in our SUMOylation assays *in vivo* and *in vitro* (data not shown). Thus involvement of SUMO-2/3 is not excluded, and the aforementioned global SUMOylation analysis was indeed based on conjugation to tagged SUMO-2^33^. Nevertheless, formation of a poly-SUMO chain on XPC seems unlikely, judging from the band pattern of SUMOylated XPC recovered from cell extracts (Fig. 1a,e). This SUMOylated XPC was present in a rather constitutive fashion, whereas UV-induced SUMOylation of XPC has been documented^27^,^30^,^31^. Notably, in the cell extracts containing XPC WT, but not XPC 3KR, a slowly migrating, minor band was visible on immunoblotting with the anti-XPC antibody (see a typical example in Fig. 2f, indicated by asterisk). Given that this band most likely represents a SUMOylated form of XPC, we would argue against the possibility that the bands observed in Fig. 1a represent co-precipitation of a certain SUMOylated protein distinct from FLAG-XPC. Although only a small fraction of XPC appears to be SUMOylated as a steady-state level, it is possible that the cycle of SUMOylation and de-SUMOylation could be so rapid that most XPC molecules may actually undergo transient SUMOylation. Since the fusion with SUMO-1 only partially alleviated the NER defect of XPC 3KR (Supplementary Fig. S4), persistent SUMOylation of XPC could be disadvantageous to achieving an optimal level of the NER activity. It would be crucial to understand how SUMOylation of XPC *in vivo* is regulated in time and space.

Our results strongly suggest that in the absence of the XPC SUMOylation, the damage handover from UV-DDB to XPC is compromised, so that UV-DDB bound to a DNA lesion blocks the following NER process (Fig. 4a). Although the XPC 3KR-expressing cells showed only a partial defect in removing 6-4PPs, one may assume that more profound effects may have been observed with repair of CPDs. In our transformed cell lines, however, CPDs were poorly repaired even with the expression of XPC WT, so that the difference from XPC 3KR was unclear. This is likely because the parental *XPC*-deficient cell line (XP4PASV) was immortalized with SV40, by which the p53 tumor suppressor was inactivated and thereby the expression levels of DDB2 were reduced. Nevertheless, the mutant XPC protein lacking the three SUMOylation sites is normally recruited to the sites with a UV-induced photolesion, and immobilized there in a UV-DDB-dependent manner (Fig. 3 and Supplementary Fig. S6). This suggests formation of a stable ternary complex containing XPC, UV-DDB and DNA, although we have not succeeded in capturing such a complex biochemically. In this situation, recruitment of the downstream NER factors, TFIIH and XPA, are compromised significantly (Fig. 2c and Supplementary Fig. S2). Biochemical and structural studies have revealed that to induce productive dual incisions by NER, XPC must interact with the undamaged DNA strand and then load the XPD helicase in TFIIH onto the damaged strand immediately 5' to the lesion^4^,^35^. Since UV-DDB directly interacts with the two affected bases comprising a photolesion^17^,^36^, it is conceivable that such persistence of UV-DDB would prevent TFIIH (and more downstream factors like XPA) from access to the lesion. It remains to be understood how SUMOylation of XPC could promote dissolution of the stalled damage recognition complexes. Since UV-induced ubiquitination of XPC is impaired by the 3KR mutations, the SUMO moiety may somehow affect activation of the CUL4 ubiquitin ligase associated with UV-DDB.

It was reported recently that DNA double strand breaks trigger the SUMOylation of multiple protein factors involved in homologous recombination repair^37^. The abrogation of SUMOylation of an individual target sites does not lead to discernable phenotypes, due to redundancy at other SUMOylation sites. The SUMOylation of XPC reported here seems to differ from this example in that the modification occurs in a rather constitutive manner and mutations at the three determined SUMOylation sites do indeed compromise NER. However, it is possible that SUMOylation of other NER proteins may also occur, resulting in modulations of their physical and functional interactions. Indeed, centrin-2^38^ and DDB2^39^ reportedly undergo SUMOylation, although the precise roles of these modifications in NER remain to be clarified. In addition, other SUMOylated proteins and/or SUMO-interacting proteins may be involved in the regulation of ubiquitination and a damage handover in NER. For example, SUMO recruits the ATP-driven, ubiquitin-specific segregase complex containing p97/VCP/Cdc48^40^,^41^, which modulates the ubiquitination of DDB2 and XPC^42^. Further studies would shed light on the molecular mechanism underlying the functional interaction between ubiquitination and SUMOylation.

## Methods

### Cell lines and cell culture

All human cell lines were cultured at 37 °C in a humidified atmosphere containing 5% CO_2_. The WI38 VA13 (normal) and XP4PASV (*XPC*-deficient) SV40-transformed human fibroblast cell lines were cultured in Dulbecco’s modified Eagle’s medium supplemented with 10% fetal bovine serum. The High Five insect cell line was cultured at 27 °C in Ex-Cell 405 medium (SAFC Biosciences).

### Establishment of stably transformed cell lines

The bicistronic mammalian expression vector pIREShyg (Takara Bio) was used for stable expression of the WT and mutant XPC proteins. Stable transformants of XP4PASV cells expressing FLAG-XPC, EGFP-XPC, or SUMO-1-XPC at nearly physiological levels were isolated as described previously^20^,^28^. The retroviral pMMP expression vector (obtained from T. Taniguchi, Fred Hutchinson Cancer Research Center, with permission from R. C. Mulligan, Harvard Medical School) was used for stable expression of HA-tagged DDB2. The pMMP construct encoding HA-DDB2 was transfected into the 293-GPG retrovirus packaging cell line using FuGENE 6 reagent (Promega). Culture supernatants containing the recombinant retrovirus (2 ml) were mixed with 1 × 10^6^ cells suspended in 8 ml of culture medium and then supplemented with polybrene at a final concentration of 8 μg/ml. After incubation at 37 °C for 6 h, the cells were transferred to normal culture medium and incubated for a further 24 h at 37 °C. Stable transformants were selected by continuous culture in the presence of 1 μg/ml puromycin (Sigma-Aldrich). To delete the endogenous *DDB2* gene, the CRISPR CD4 nuclease vector (Life Technologies) was used. Double-stranded oligonucleotides encoding the CRISPR RNA target site within exon 1 of the human *DDB2* gene (5´-GGGGCGTAATACAATATCGGAGG-3´) were cloned into the vector, and the resulting construct was introduced into the cell lines stably expressing FLAG-XPC (WT or the 3KR mutant) using Lipofectamine 2000 reagent (Life Technologies). CD4-positive cells were selected with Dynabeads CD4 (Life Technologies), and sparsely seeded into culture dishes to allow isolation of single colonies. Depletion of DDB2 was checked by immunoblotting for each clone.

### Preparation of cell lysates

For immunoblot and pull-down experiments, cells (typically in 60 mm dishes) were washed twice with ice-cold phosphate-buffered saline and then lysed on ice for 60 min with 500 μl of NP lysis buffer [25 mM Tris-HCl (pH 8.0), 1 mM EDTA, 10% glycerol, 1% Nonidet P-40, 0.25 mM phenylmethylsulfonyl fluoride, and Complete protease inhibitor cocktail (Roche Diagnostics)] containing 0.3 M NaCl and 20 mM *N*-ethylmaleimide. The cell lysates were scraped into microcentrifuge tubes and the dishes were washed with 500 μl of the same buffer, which was combined with the recovered lysates. Soluble extracts were obtained by centrifugation for 10 min at 20,000 x g.

### Preparation of recombinant proteins

The heterodimeric complex containing FLAG-XPC (WT or mutant) and RAD23B-His was purified as described previously^43^. The DDB1-DDB2 heterodimer and DDB1-DDB2-CUL4A-RBX1 heterotetramer (CRL4^DDB2^) were also purified as described previously^20^. For pull-down experiments, a recombinant baculovirus that co-expressed non-tagged DDB1 and DDB2 fused to a FLAG-tag and AviTag in tandem (FA-DDB2) was generated using the pFastBac Dual vector (Life Technologies). High Five cells were infected with this baculovirus and the DDB1/FA-DDB2 complex was purified as described previously^20^. To prepare the biotinylated UV-DDB complex, the purified DDB1/FA-DDB2 complex was immobilized on Heparin Sepharose 6 Fast Flow beads (GE Helthcare Biosciences) and biotinylated *in vitro* with biotin ligase (Avidity), as described previously^44^. The other human NER proteins, including centrin-2, TFIIH, FLAG-XPA, RPA, ERCC1-His/XPF, and XPG, were also purified as described previously^35^,^43^. To prepare the recombinant SUMO-1 protein, a HA-tag and a hexahistidine (His)-tag were fused in tandem to the N-terminus of the SUMO-1 sequence and cloned into the pET-28a vector (Novagen). The His-HA-SUMO-1 protein was expressed in the *Escherichia coli* BL21 (DE3) strain and then purified using a HisTrap FF column (GE Healthcare Biosciences). The E1 (SAE1/UBA2) and E2 (UBE2I) enzymes used for SUMOylation were purchased from Boston Biochem.

### *In vitro* SUMOylation and ubiquitination assays

The standard reaction mixture (15 μl) for SUMOylation contained 50 mM Tris-HCl (pH 7.5), 5 mM MgCl_2_, 0.2 mM CaCl_2_, 70 mM NaCl, 2 mM ATP, 0.2 mM dithiothreitol, bovine serum albumin (1.5 μg), E1 (0.25 μg), E2 (0.5 μg), His-HA-SUMO-1 (0.5 μg), and the FLAG-XPC/RAD23B-His complex (10 ng). The reactions were incubated at 30 °C for 30 min, stopped by the addition of 1 μl of 0.5 M EDTA, and then subjected to SDS-PAGE followed by immunoblot analyses using the appropriate antibodies. *In vitro* ubiquitination assays with the purified CRL4^DDB2^ complex were performed as described previously^20^.

### Immunoprecipitation from cell extracts

The pCAGGS vector was used for transient overexpression of HA-tagged SUMO-1^45^. The HA-SUMO-1 expression construct was transfected into FLAG-XPC-expressing XP4PASV cells in 60 mm culture dishes using Lipofectamine 2000 reagent (Life Technologies). Where indicated, the FLAG-XPC and HA-SUMO-1 constructs were also co-transfected into XP4PASV cells. Soluble cell extracts were prepared as described above and then incubated with 50 μl of anti-FLAG M2 affinity gel (Sigma-Aldrich) at 4 °C overnight. The beads were washed seven times with NP lysis buffer containing 0.3 M NaCl, followed by seven times with NP lysis buffer containing 1 M NaCl, and then once with NP lysis buffer containing 0.3 M NaCl. The bound proteins were eluted with 50 μl of NP lysis buffer containing 0.3 M NaCl and 500 μg/ml of the FLAG peptide.

### Immunoblotting

In immunoblot analyses, the secondary antibodies were conjugated to alkaline phosphatase (Sigma-Aldrich) or horseradish peroxidase (R&D Systems) and detected using CDP-Star (Roche Diagnostics) or Immobilon Western (Millipore), respectively. Images were collected using an ImageQuant LAS 4010 biomolecular imager (GE Healthcare Biosciences) and by exposure to RX-U X-ray films (Fujifilm). Quantifications were performed using ImageQuant TL (GE Healthcare Biosciences) software.

### Measurement of the *in vivo* repair rates of UV-induced 6-4PPs

The cells were cultured to 80% confluence in 100 mm dishes and then incubated at 37 °C for 2 h in medium containing 6 mM thymidine to prevent the dilution of DNA damage by replication. The cells were then irradiated with UVC (10 J/m^2^) under a germicidal lamp with a 254 nm peak (GL-15; Toshiba) and cultured in the presence of 6 mM thymidine for various times to allow DNA repair. Genomic DNA was purified using the QIAamp DNA Blood Mini Kit (Qiagen) and the level of remaining 6-4PP was determined by an enzyme-linked immunosorbent assay using a lesion-specific monoclonal antibody (64M-2: Cosmo Bio), as described previously^46^.

### Local UV irradiation

Local UV irradiation through isopore membrane filters and immunofluorescence staining were carried out as described previously^47^. Subnuclear regions exposed to UV were visualized with anti-CPD monoclonal antibody (TDM-2: Cosmo Bio)^46^. Accumulation of EGFP-XPC at local UV-damaged areas was analyzed using a customized fluorescence microscopy system based on the IX71 inverted microscope (Olympus). Briefly, deep UV light (250 ± 5 nm) emitted from an Hg-Xe lamp was focused within the cell nucleus using a reflecting objective lens (OBLR-20A: Sigma Koki) equipped at the upright position of the microscope. The focus was first adjusted under the microscope with visible light from a halogen lamp, and the optical path was then switched to UV through the U-MBF3 mirror unit (Olympus). After the focused UVC light was applied to a certain area within the nucleus for 5 sec, time-lapse images of EGFP were collected every 2 sec for a total of 58 sec through an objective lens at the inverted position. MetaMorph software (Molecular Devices) was used to control the electromagnetic shutter for UV irradiation and the cooled CCD camera for imaging, and to analyze data.

### Fluorescence recovery after photobleaching (FRAP)

One day prior to each experiment, cells stably expressing EGFP-XPC were seeded onto 35 mm glass bottom dishes (MatTek). FRAP was performed under a controlled environment at 37 °C with a CO_2_ supply using the TCS SP5 confocal microscope (Leica) equipped with Hybrid detector (Leica). A strip of area spanning the nucleus was photobleached for 453 msec at the maximum power of the 488 nm Ar laser line. Recovery of the fluorescence intensity in the bleached area was monitored every 151 msec for a total of 25 sec at 10% of the bleaching intensity. The relative fluorescence intensity was calculated at each time point and plotted as described previously^28^. Where indicated, mouse DDB2 fused to mCherry (mCherry-mDDB2) was transiently expressed as described previously^28^. At 24 h post-transfection, cells from the same dishes with and without mCherry-mDDB2 expression (discriminated by the presence of red fluorescence) were subjected to FRAP.

### NER dual incision assay

An internally ^32^P-labeled, double-stranded circular DNA substrate containing a site-specific 6-4PP was prepared as described previously^5^. Using this substrate, the cell-free NER dual incision reaction was reconstituted with purified recombinant proteins as described previously^44^. After incubation at 30 °C for 1 h, the DNA was purified and subjected to 10% denaturing PAGE followed by autoradiography.

### DNA-binding assays

Electrophoretic mobility shift assays were performed essentially as described previously^20^. DNA-binding assays with paramagnetic beads were also performed as described previously^20^.

### Pull-down assay

The biotinylated UV-DDB complex (60 ng) was incubated on ice for 1 h with 0.2 mg of streptavidin-coated paramagnetic beads (Dynabeads M-280 Streptavidin: Life Technologies) in buffer containing 25 mM Tris-HCl (pH 7.5), 1 mM EDTA, 0.1 M NaCl, 10% glycerol, 1 mM dithiothreitol, 0.01% Triton X-100, and 0.25 mM phenylmethylsulfonyl fluoride. After removal of the unbound proteins by washing four times with the same buffer, the beads were incubated on ice for 1 h with the XPC/RAD23B/centrin-2 complex (30 ng) in the same buffer. The beads were then washed a further five times with the same buffer and subjected to SDS-PAGE followed by immunoblot analyses using the appropriate antibodies.

### Antibodies

The anti-XPC^35^ and anti-RAD23B^48^ antibodies, used for immunoblotting, were obtained as described previously. For immunofluorescence staining, another anti-XPC antibody raised against the full-length XPC protein^48^ as well as anti-XPB (S-19) and anti-XPA (FL-273) antibodies (both purchased from Santa Cruz Biotechnology) were used. The anti-DDB1, anti-DDB2, and anti-HA (3F10) antibodies were purchased from BD Transduction Laboratories, R&D Systems, and Roche Diagnostics, respectively.

## Additional Information

**How to cite this article**: Akita, M. *et al.* SUMOylation of xeroderma pigmentosum group C protein regulates DNA damage recognition during nucleotide excision repair. *Sci. Rep.* **5**, 10984; doi: 10.1038/srep10984 (2015).

## Supplementary Material

Supplementary Information
