# Supplementary material for: SUMOylation of xeroderma pigmentosum group C protein regulates DNA damage recognition during nucleotide excision repair
Source: Sci Rep. 2015 Jun 4;5:10984. doi: 10.1038/srep10984 (PMC4455304; doi:10.1038/srep10984)
Supplement: Supplementary Information [file srep10984-s1.pdf]

SUPPLEMENTARY INFORMATION

SUMOylation of xeroderma pigmentosum group C protein regulates DNA damage recognition during nucleotide excision repair

Masaki Akita[1], Yon-Soo Tak[1], Tsutomu Shimura[1], Syota Matsumoto[1,2], Yuki Okuda-Shimizu[3], Yuichiro Shimizu[3], Ryotaro Nishi[1,3], Hisato Saitoh[4], Shigenori Iwai[5], Toshio Mori[6], Tsuyoshi Ikura[7], Wataru Sakai[1,2], Fumio Hanaoka[3,8] & Kaoru Sugasawa[1,2,3]

[1]Biosignal Research Center, Organization of Advanced Science and Technology, Kobe University, Kobe 657-8501, Japan, [2]Graduate School of Science, Kobe University, Kobe 657-8501, Japan, [3]Cellular Physiology Laboratory, RIKEN, Wako 351-0198, Japan, [4]Graduate School of Science and Technology, Kumamoto University, Kumamoto 860-8555, Japan, [5]Graduate School of Engineering Science, Osaka University, Toyonaka 560-8531, Japan, [6]Radioisotope Research Center, Nara Medical University, Kashihara 634-8521, Japan, [7]Radiation Biology Center, Kyoto University, Kyoto 606-8501, Japan, [8]Faculty of Science, Gakushuin University, Tokyo 171-8588, Japan.

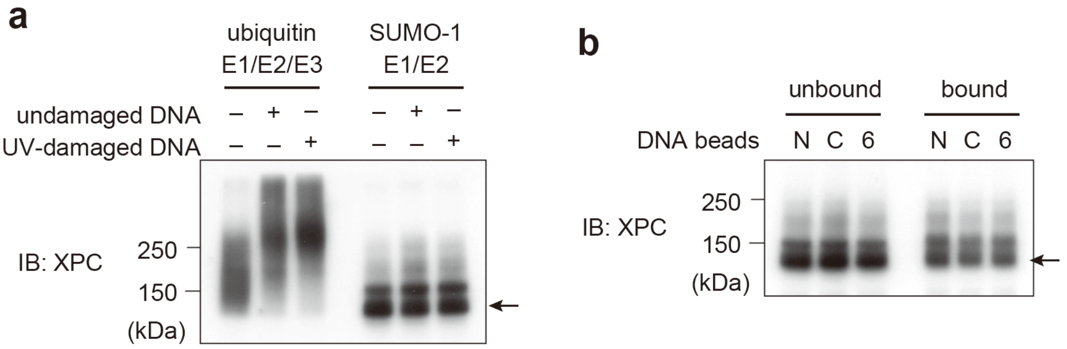

Supplementary Figure S1 | XPC SUMOylation is not stimulated by DNA and does not affect its DNA-binding activity. (a) Immunoblot analyses of XPC from *in vitro* ubiquitination and SUMOylation reactions performed in the presence or absence of undamaged or UV-damaged DNA. (b) Immunoblot analyses of XPC from *in vitro* SUMOylation reactions performed in the presence of paramagnetic beads on which DNA containing no damage (N), cyclobutane pyrimidine dimers (C), or 6-4PP (6) was immobilized. After incubation, the proteins that were bound and unbound to the DNA beads were separated and subjected to immunoblot analyses. The arrows indicate unmodified XPC.

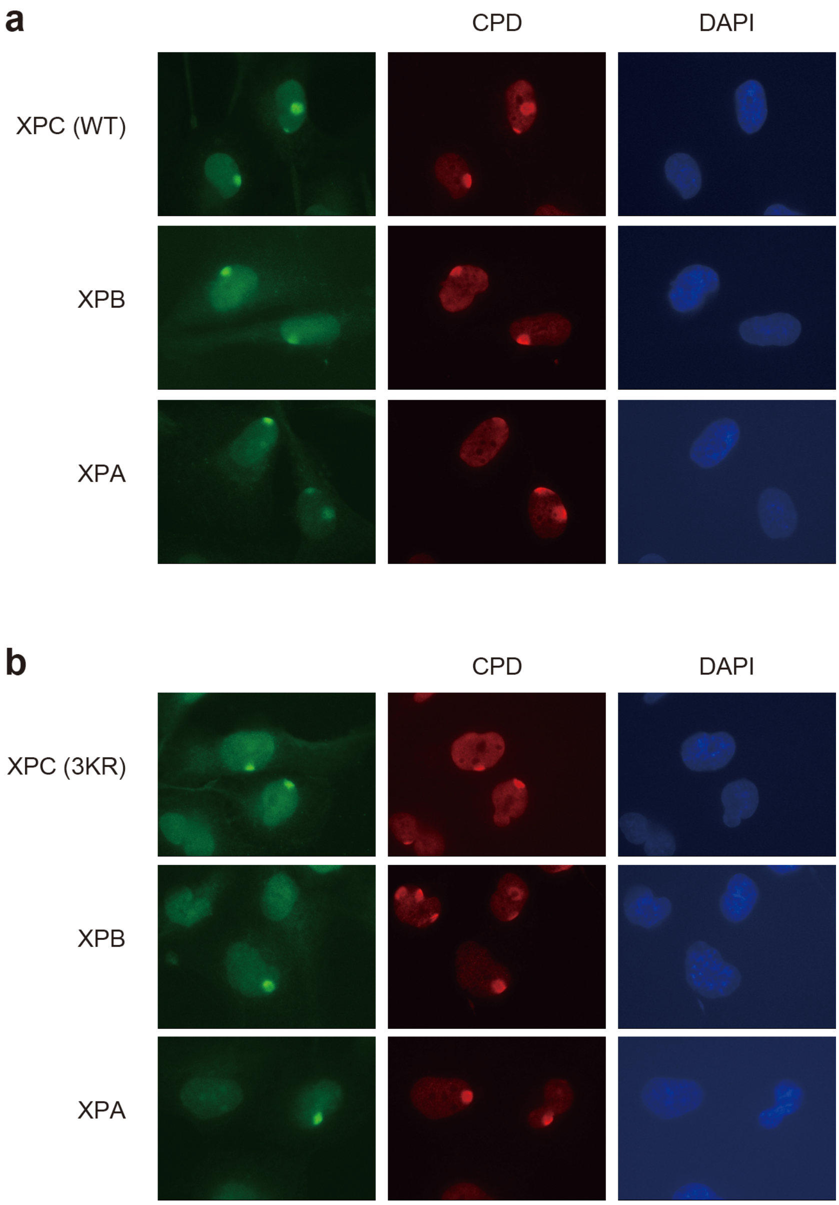

Supplementary Figure S2 | The XPC 3KR mutant is normally recruited to the sites with UV-induced photolesions but compromises recruitment of the downstream NER factors. The transformed XP4PASV cell lines stably expressing the XPC WT (a) or 3KR (b) protein were irradiated with UVC (at 100 J/m^2) through isopore membrane filters. At 30 min post irradiation, the cells were subjected to immunofluorescence staining with antibodies against the indicated NER proteins (green). The presence of UV-induced CPD was also visualized (red), and nuclei were counterstained with DAPI (blue).

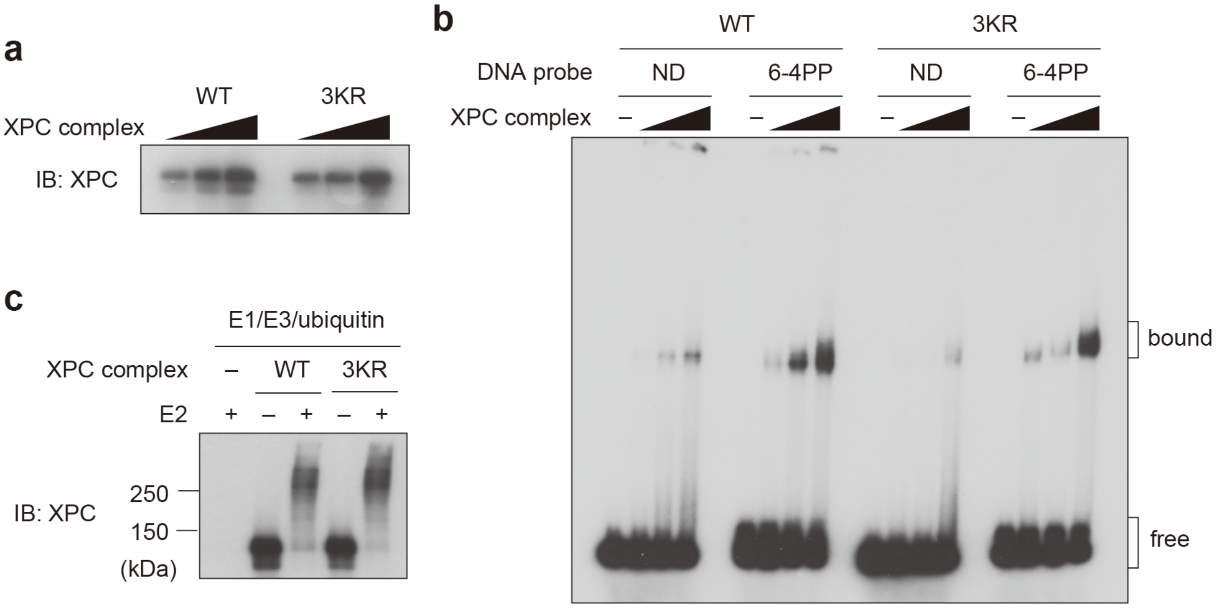

Supplementary Figure S3 |The XPC 3KR mutant protein exhibits normal damage recognition activity and CRL4$^{\mathbf{DDB2}}$**-mediated ubiquitination *in vitro*.** (a) Immunoblot analyses of the XPC WT and 3KR protein complexes confirming that comparable amounts of proteins were included in the electrophoretic mobility shift assays. (b) Electrophoretic mobility shift assays using the XPC WT or 3KR complex and DNA probes (approximately 180 bp) containing no damage (ND) or a single 6-4PP at a specific site. The positions of the free DNA and XPC-DNA complex (bound) are indicated. (c) Immunoblot analyses of XPC WT and 3KR in cell-free ubiquitination reactions performed in the presence (+) or absence (-) of E2.

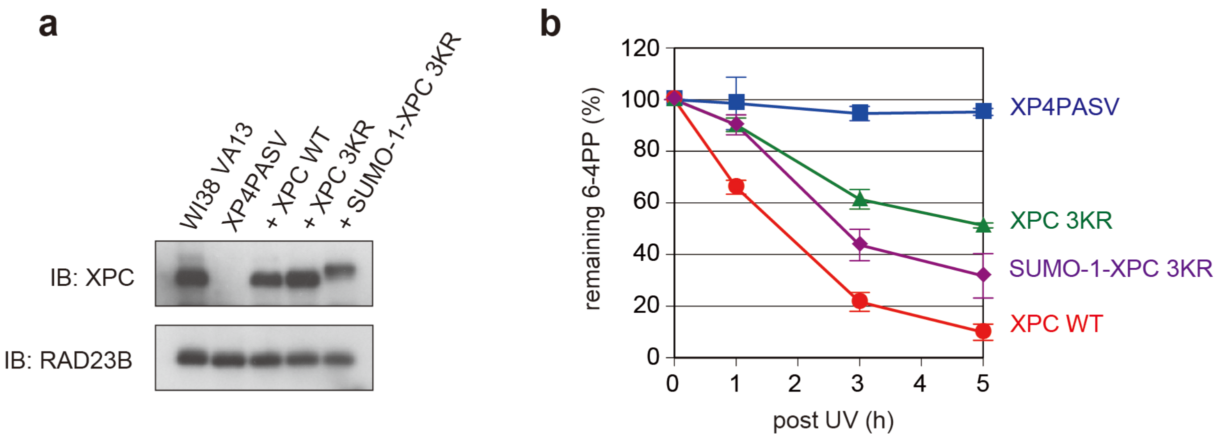

Supplementary Figure S4 | SUMO-1 fusion partially alleviates the NER defect caused by the XPC 3KR mutations. (a) Immunoblot analyses comparing the levels of the ectopically expressed XPC proteins. RAD23B was detected on the same blot as a loading control. (b) DNA repair assays of the indicated cell lines following exposure to UVC (10 J/m^2). The percentages of 6-4PP remaining in the genomic DNA were quantified and plotted as a function of time. The mean values and standard errors were calculated from two independent experiments.

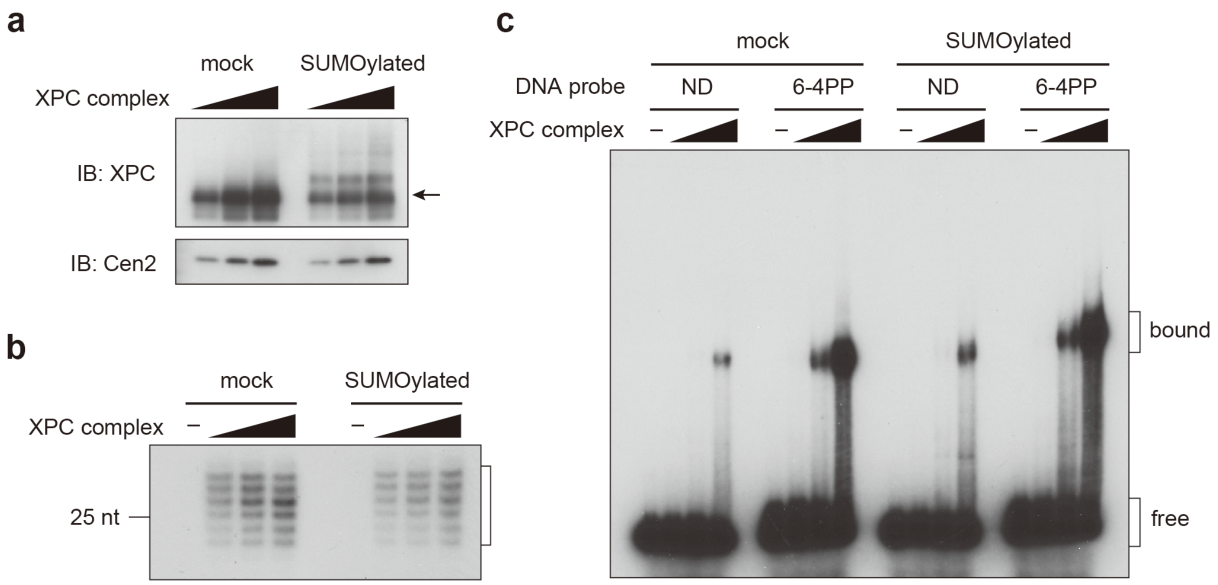

Supplementary Figure S5 | The effects of SUMOylation on NER and the DNA damage recognition activity of the XPC protein *in vitro*. (a) Immunoblot analyses of the recombinant FLAG-XPC WT protein bound to RAD23B-His and centrin-2 (Cen2) and then immobilized on anti-FLAG M2 affinity beads and subjected to *in vitro* SUMOylation reactions. For the mock treatment, a similar reaction was carried out in parallel without E1. After an extensive wash, the proteins were recovered by incubating with the FLAG peptide, and varying amounts were subjected to immunoblot analyses, which confirmed that comparable amounts of proteins were included in the following assays. (b) Cell-free NER dual incision assays using increasing amounts of the protein complexes shown in (A). The excised oligonucleotides containing a 6-4PP are indicated. (c) Electrophoretic mobility shift assays using SUMOylated and unmodified XPC WT proteins and DNA probes containing no damage (ND) or a single 6-4PP at a specific site. The positions of the free DNA and XPC-DNA complex (bound) are indicated.

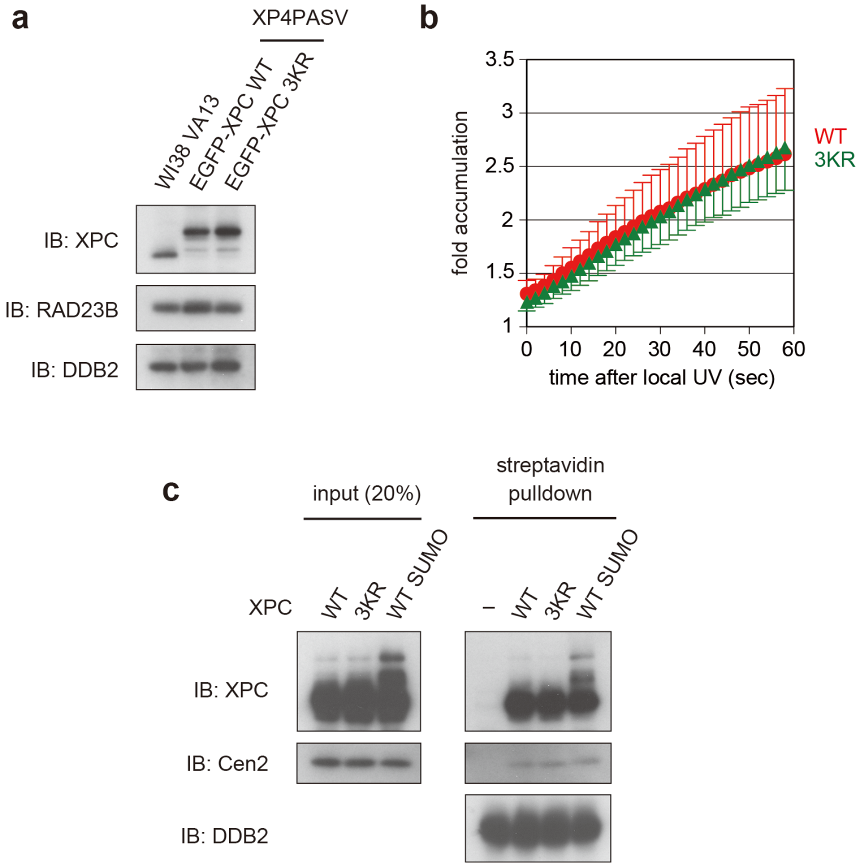

Supplementary Figure S6 | XPC SUMOylation does not affect its recruitment to local UV damage or physical interaction with UV-DDB. (a) Immunoblot analyses of the transformed XP4PASV cells stably expressing EGFP-tagged XPC WT or 3KR. The expression levels of RAD23B and DDB2 in the same samples were measured as controls. (b) Time-lapse images of the cells stably expressing EGFP-tagged XPC WT or 3KR were obtained after a pulse of focused deep UV light was applied to a certain area within a cell nucleus. For each image, the relative fluorescence intensity of the irradiated area relative to that of a non-irradiated area within the same nucleus was plotted as a function of time. The mean values and standard errors were calculated from analyses of 20 cells per condition. (c) The UV-DDB complex containing biotinylated DDB2 was immobilized on streptavidin-coated paramagnetic beads and used to pull down the purified XPC/RAD23B/centrin-2 heterotrimeric complex containing XPC WT, SUMOylated XPC WT, or XPC 3KR protein. The bound proteins and input samples were subjected to immunoblot analyses using the indicated antibodies.

```
                                                                                                                  81          89
H.sapiens     ---------- ----MARKRA AGGEPRGREL RSQKSKAKSK ARREEEEEDA FEDEK--PPK KSLLSKVSQG KRKRGCSHPG GSADGPAKKK VAKVTVKSE- -----NLKVI KDEALSDGDD
M.musculus    ---------- ----MAPKRT ADGR---RRK RGQKTEDNKV ARHEESVADD FEDEKQKPRR KSSFPKVSQG KRKRGCSDPG DPTNGAAKKK VAKATAKSK- -----NLKVL KEEALSDGDD
G.gallus      ---------- ----MARKRK ASVPRAPAGK RRPGGAGVRR EREEEREEGD FVEEK--RSV KKSRAGVAAW KEDDGGAGAA KSANTPSKQK GVKRQAKENE ISPENKGTHH KEQTCSDSRK
D.rerio       ---------- ---------- --------MA KRKDTRQKSD TKKPKQIANT KSGSKTQKAK ENGMETKKNL KNSKVASRRS RKVKDVLDEV TSKYFQDSE- ------VKTE EPEDLSDHSE
A.thaliana    ---------- ---------- -----MKSRS ESKNCRLAQA SRVAVNKVLD KSSARGSRGK KKQDDNCDSA KRDKGVNGKG KQALDARLID NVLEDRGCG- ---------- ----------
C.elegans     METRRRSSRL QQQTTSDLNA REEPQPSEPP VKRARGAKKN EAPSTAPMPP KTTKKSTKPS RKQGSPIEAE LDEMGFELEN EEEAEIVVQK SKKNGRKLVK IDENLRISAE NGSKSSNFLE
                                                                          :      ..           .

                                                                                                                       183
H.sapiens     LRDFPSDLKK AHHLKRGATM NEDSNEEEEE SENDWEEVEE L-----SEPV ---------- -LGDVRESTA FSRSLLPVKP VEIEIETPEQ AKTRERSEKI KLEFETYLRR AMKRFNKGVH
M.musculus    FRDSPADCKK AKKHPKSKVV DQG--TDEDD SEDDWEEVEE L-----TEPV ---------- -LDMG-ENSA TSPSDMPVKA VEIEIETPQQ AKERERSEKI KMEFETYLRR MMKRFNKEVQ
G.gallus      APRKEIKLKR ESPIKKEMDE DNTDDDDDDE SEDEWEDVEE L-----QEPA ---------- -TDKLEQGAV LPAVVLPSNP VEIEIETPEQ LKKRERREKR KAEFETYVRR MIKRFTKEVR
D.rerio       ERMIIEDTSL SKQVKEE--- -----EEDSE DEDDWEEVEE M-----AGPL GPVDSSELAL E--------- -------SKP VEIEIETPDM IRKRQKKEKR KAEFETYLRR MMNRFNKDLL
A.thaliana    ---------- ---------- ---NVDDDEM NDSDWEDCPI P-----SLDS ---------- ---------- ---------T VDDNNVDDTR ELTIEFDDDV PDAKKQKNAY RATAEDKVRA
C.elegans     ENRMEIDEKP GNLAKKSSKN GSRVVKSDEK SENLVQSVPK STTNGS---- ---------- -KVAIIEDDP EIRAENGVKS SKSDEKPDFS AQNGSKLAQN APNRISRPRR SVTTAKKVSY
                                           .:. .:.  :.                                               .  . :           .   .               .   *

H.sapiens     EDTHKVHLLC LLANGFYRNN ICSQPDLHAI GLSIIPARFT RVL-PRDVDT YYLSNLVKWF IGTFTVNAEL SASEQDNLQT TLERRFAIYS ARDDEELVHI FLLILRALQL LTR-------
M.musculus    ENMHKVHLLC LLASGFYRNS ICRQPDLLAI GLSIIPIRFT KVP-LQDRDA YYLSNLVKWF IGTFTVNADL SASEQDDLQT TLERRIAIYS ARDNEELVHI FLLILRALQL LTR-------
G.gallus      EDTHKVHLLC LLANGFYRNR ICSQPDLHAI GLSIIPIHFT KVP-AGQVDL LYISNLVKWF VGTFTVNDEL STEKGEPLQS TLERRFAIYA ARDDEELVHI FLIILRALQL LCR-------
D.rerio       VDTHKVHLLC LMASGLFRNR LLCEPDLLAV ALSLLPSHFT TVS-LKRINN GFLEGLLKWF QATFTLNPAL PEEKEVDLRT VLEKRMGCLS ARNHEEMTYL FLLVLRSLRL FCR-------
A.thaliana    ELVHKVHLLC LLARGRIVDS ACNDPLIQAA LLSLLPSYLT KVSNLEKVTV KDIAPLLRWV RENFSVSCSP SSEKS--FRT SLAFALESRK G-TAEELAAL AVALLRALKL TTR-------
C.elegans     VPSDDQLELS SSSSELESSS EDEDTEIRPK TGSKIAKKRE KSFKISESES SSESPDDESE ASEASEDPSI PGPSEPRKRR KIQRKSTLSS GGATTKDLHW PKCSKASIAR KTGNPGSKNA
                  ..  *.    :     .    :. : .     *:.                        .       : .    .  .    :   :         .    :           ::

H.sapiens     ----LVLSLQ PIPLKSATAK GKKPSKERLT ADPGGSSETS SQVLENHTKP KTSKGTK--- -----QEETF AKGTCRPSAK GKRNKGGRKK RSKPSSSEED EGPGDKQEKA TQRRPHGRER
M.musculus    ----LVLSLQ PIPLKSAVTK GRKSSKETSV EGPGGSSELS SNSPESHNKP TTSRRIK--- -----EEETL SEGRGKATAR GKRGTGTAGS RQRRKPSCSE ---GEEAEQK VQGRPHARKR
G.gallus      ----LVLSLQ PIPLKETKAK EKSTSKKQSL SSTSEGQESS GTTPKAVAKK CPCKKAKRDE KSSGSEEDNE ESKKTKSAQT ERTHKSKLTA GNREQKETRN VDSGLREKDV PVRPKNSRWR
D.rerio       ----LVLSLQ PLPLKPPPAT KSKTTPSKSS SEKAQSEKSS P--------- ---------- ---------- ELKVSPGSKR PSSATAAAKE DRGGKRKKKT GGGGDKEAAG AQKPKNSRRR
A.thaliana    ----FVSILD VASLKPGADR NESSGQNRAK MKHG------ ---------- ---------- ---------- -IFRTSTLMV PKQQAISSYP KKSSSHVKNK SPFEKPQLGN PLGSDQVQDN
C.elegans     AKKPAKSSLR MAQKQKPDQP WKKNLKDYET DRKLAKGERR MLEYRHVAHA YVARGKIEEI TYDEAYKLHE MMKKAYFAGR SLLDAAVLDP VEFEKSQKNV KKSEKNDEKN TAGDSSSSED
                      *      :        .   .                                                                           .

H.sapiens     RVASRVSYKE ESGSDEAGSG SDFELSSGEA SD-------- ---------- ---------- ---------- ---------- ---------- ---------- ---------- ----------
M.musculus    RVAAKVSYKE ESESDGAGSG SDFEPSSGEG QH-------- ---------- ---------- ---------- ---------- ---------- ---------- ---------- ----------
G.gallus      RVASKVCYKE ESGSD-EGSV SDFEIS-GEE SD-------- ---------- ---------- ---------- ---------- ---------- ---------- ---------- ----------
D.rerio       SVASKVSYK- EVGSEEEEEQ SEEEFQPSNE DD-------- ---------- ---------- ---------- ---------- ---------- ---------- ---------- ----------
A.thaliana    AVNSSCEAGM SIKSDGTRRK GDVEFERQIA MA-------- ---------- ---------- ---------- ---------- ---------- ---------- ---------- ----------
C.elegans     EWEEMEHFQP PIIDDNIEVS IDHEGGGGDD GEEVVKDWWA IYLRQEINRK IREMWENTHK VHLLCFMAHL KFVVKIALDE SLVPSLMMSQ LPNGYLKFIG EPVVPIDIMK NLVKWFADAF
                            .:       : *

H.sapiens     ---------- ---------- ---------- ---------- ---------- ---------- -PSDEDSEPG PPKQRKAPAP QRTKAGSKSA SRTHRGSHRK DPSLPAASSS SSSSKRGKKM
M.musculus    ---------- ---------- ---------- ---------- ---------- ---------- -SSDEDCEPG PRKQKRASAP QRTKAGSKSA SKTQRGSQCE PSSFPEASSS SSGCKRGKKV
G.gallus      ---------- ---------- ---------- ---------- ---------- ---------- -ISDEDFETV SKKRRSSQGA QKSKVMTVKS PKSETSESRL SRNSLGVEPR PHAQRKRNKI
D.rerio       ---------- ---------- ---------- ---------- ---------- ---------- -SEDSDGAVK ICRKSKVKSR RSSKVKQEER SEEEEEEEEE EEEEKEVKKQ RRKKKQGK--
A.thaliana    ---------- ---------- ---------- ---------- ---------- ---------- -LSATADNQQ SSQVNNTKKV REITKISNSS SVSDQVISTA FGSKKVDSP- ----------
C.elegans     RPLNGVVSVA SIEQDSLLEG HEARYPETRR LTALVDAKCF ETDLDRATLL FCLLRGLELT TRLVVNVRAI PRRWDKTQQK ELQNELSKFR ELSRSRSTTP GEKSLEIQEK SGEKSQKPAK
                                                                                      :        .  .                    .    .

H.sapiens     CSDGEKAEK- --RSIAGIDQ WLEVFCEQE- --EKWVCVDC VHGVVG--QP LTCYKYATKP MTYVVGIDSD GWVRDVTQRY DPVWMTVTRK CRVDAEWWAE TLRPYQ---- ----------
M.musculus    SSGAEEMAD- --RKPAGVDQ WLEVYCEPQ- --AKWVCVDC VHGVVG--QP VACYKYATKP MTYVVGIDSD GWVRDVTQRY DPAWMTATRK CRVDAEWWAE TLRPYR---- ----------
G.gallus      ISSDEDDGQQ MVRKVVGTDQ WLEVFLERE- --DRWVCVDC VHGIVG--QP QQCFTYATKP LSYIVGFDND GSVKDVTQRY DPVWMTMTRK KRVDPEWWED TLQPYK---- ----------
D.rerio       ---------- ------GADE WLEVYLESS- --GRWVCVDV DQGVG---QP QLCSDQATLP ITYVVGLDDE GFMKDLGSRY DPTWLTSSRR RRVDSEWWEE TMELYK---- ----------
A.thaliana    ---------- --------LC WLEVYCNGEN MDGKWVHVDA VNGMIDAEQN IEAAAAACKT VLRYVVAFAA GGAKDVTRRY CTKWHTISSK -RVSSVWWDM VLAPLVHLES GATHDEDIAL
C.elegans     KGQKSEKKAA KKVVVEERNY WVEYWQPRE- --KRWICVDP LHKSVD--EP LSIHEHSASP ISYVFAIDNK QGICEVSQRY AMDCVKQDFR RRRTNPKWVA WTLFLP---- ----------
                                    *:* :    .    :*: **    :      :        :  . :   .          ::  **      .   :  *     *

                                                                655
H.sapiens     ---------- -------SPF MDREKKEDLE FQAKHMDQPL PTAIGLYKNH PLYALKRHLL KYEAIYPETA AILGY--CRG EAVYSRDCVH TLHSRDTWLK KARVVRLGEV PYKMVKGFSN
M.musculus    ---------- -------SLL TEREKKEDQE FQAKHLDQPL PTSISTYKNH PLYALKRHLL KFQAIYPETA AVLGY--CRG EAVYSRDCVH TLHSRDTWLK QARVVRLGEV PYKMVKGFSN
G.gallus      ---------- -------SPF VDRDKKEETE FQVKLQDQPL PTAIGEYKNH PLYALKRHLL KYQAIYPESA AILGY--CRG EAVYSRDCVH TLHSKDTWLK QARVVRIGEV PYKMVKGYSN
D.rerio       ---------- -------SPD TERGQKEDQE MQAKLLDKPL PTSVSEYKNH PLYVLKRHLL KYEALYPATA AVLGY--CRG EPVYSRDCVH TLHSRDTWLK EARTVRLGEE PYKMVLGFSN
A.thaliana    RNFNGLNPVS SRASSSSSSF GIRSALEDME LATRALTESL PTNQQAYKSH EIYAIEKWLH KNQILHPK-G PVLGF--CSG HPVYPRTCVQ TLKTKERWLR DGLQLKANEV PSKILKRNSK
C.elegans     ---------- -----PFAAN SERKKWEMMQ MREDLVKRPL PTVMSEYKNH PLYALEKDLL KFEAIYPPPA TQKPLGQIRG HNVYPRSTVF TLQGENNWLK LARSVKIGEK PYKIVKARPD
                                :     *   *  : :      ..* **    **.*  :*.:::  *  * : ::*  . .        * .**.*   *  **: .: **:  .  ::  .*  *  *::   ..

H.sapiens     RARKARLAEP QLREEND--- LGLFGYWQTE EYQPPVAVDG KVPRNEFGNV YLFLPSMMPI GCVQLNLPNL HRVARKLDID CVQAITGFDF HGGYSHPVTD GYIVCEEFKD VLLTAWENEQ
M.musculus    RARKARLSEP QLHDHND--- LGLYGHWQTE EYQPPIAVDG KVPRNEFGNV YLFLPSMMPV GCVQMTLPNL NRVARKLGID CVQAITGFDF HGGYCHPVTD GYIVCEEFRD VLLAAWENEQ
G.gallus      QARKARLAEP ANRDKAD--- LALFGRWQTE EYQPPIAVDG KVPRNEYGNV YLFLPSMLPI GCVQLRLPNL NRLARKLDID CAQAVTGFDF HGGYSHAVTD GYVVCEEYKE VLIAAWENEQ
D.rerio       RSRKARMMSE Q-KNVKD--- LALFGTWQTE EYQPPIAVDG KVPRNEFGNV YMFKSCMLPI GCVHVHLPNL HRVARKLNID CALAVTGFDY HCGFAHAVND GYIVCEEHEE ILKAAWENEQ
A.thaliana    FKKVKDFEDG DNNIKGGSSC MELYGKWQME PLCLPPAVNG IVPKNERGQV DVWSEKCLPP GTVHLRFPRI FAVAKRFGID YAPAMVGFEY RSGGATPIFE GIVVCTEFKD TILEAYAEEQ
C.elegans     PRIPVEDRED KFLDVYG--- -----YWQTE KYRRPPLKNG KIPHNEYGNV YMFNENMCPL DCTYLKLSGL VQISRKLGKQ CIPAVVGWAF DGGFTHPVID GAIVLEKDAI DFINAWEKLE
                      .        .          ** *     *   :*  :*:** *:*  ::     *  . . : :. :   :::::. :    *:.*: :   *   .: : * :* :      :  *: : :

H.sapiens     AVIERKEKEK KEKRALGNWK LLAKGLLIRE RLKRRYGPKS EAAAP----- ---HTDAGGG LSSDEEEGTS SQAEAARILA ASWPQNREDE EKQKLKGGPK KTKREKKAAA SHLFPFEQL-
M.musculus    AIIEKKEKEK KEKRALGNWK LLVRGLLIRE RLKLRYGAKS EAAAP----- ---HAAGGG- LSSDEEEGTS SQAEAARVLA ASWPQNREDP EQK--SEYTK MTRKRRAAEA SHLFPFEKL-
G.gallus      AEIEKKEKEK REKRALGNWK LLTKGLLIRE RLKQRYSIKV EPSAP----- ---ETEKGGG FSSDEEGPSS GTAGGG--ME IFWPRNRQAE KQK--EKATR KSKQEKKEEA AQLFPFEKL-
D.rerio       EIQQKKEQEK REKRAVTNWT LLVKGLLIKE RLKRRYGQQG LASGTGLKQG --KEGGAEGL SSDEEKEGGA QSAQAPPSLT ASWPQNRKEE AEEK--IVRR VSNREKRGEE KHLFPFEKV-
A.thaliana    EKKEEEERRR NEAQAASRWY QLLSSILTRE RLKNRYANNS NDVEAKSLEV NSETVVKAKN VKAPEKQRVA KRGEKSRVRK SRNEDESHEH VFLDEEETFD EETSVKTKRC KCGFSVEVEQ
C.elegans     SGRAEKEEKQ RVEKIHENWK KLIKGMLRLA YVRKQFGHPA AEKPTK---- --RQKIGGKT EEIEENEGGA GPPSSVDHIT DNTEQITPMH GFSLDFINKK K--------- ----------
                  .:*..: .  :   .*   *  .:*     :: ::.        .                  .  *:   :

H.sapiens     -
M.musculus    -
G.gallus      -
D.rerio       -
A.thaliana    M
C.elegans     -
```

Supplementary Figure S7 | Evolutionary conservation of the XPC SUMOylation motifs. Amino acid sequences of the XPC orthologs from indicated species are aligned by ClustalW, in which putative SUMOylation motifs predicted by the SUMOplot analyses are highlighted. The motifs with high probability (score 0.9 or higher) are shown in red, whereas those with moderate probability (higher than 0.65 and lower than 0.9) are in blue. The four SUMOylation motifs in human XPC are numbered. Accession numbers of the reference sequences used are as follows: *H. sapiens*, NP_004619.3; *M. musculus*, NP_033557.2; *G. gallus*, XP_414379.3; *D. rerio*; NP_001038675.1; *A. thaliana*, NP_197166.2; *C. elegans*, NP_500156.2.
